# A Deep Learning Framework for the Characterization of Thyroid Nodules from Ultrasound Images Using Improved Inception Network and Multi-Level Transfer Learning

**DOI:** 10.3390/diagnostics13142463

**Published:** 2023-07-24

**Authors:** O. A. Ajilisa, V. P. Jagathy Raj, M. K. Sabu

**Affiliations:** 1 Department of Computer Applications, Cochin University of Science and Technology, South Kalamassery, Kochi 682022, Kerala, India; 2 School of Management Studies, Cochin University of Science and Technology, South Kalamassery, Kochi 682022, Kerala, India

**Keywords:** inception, squeeze-excitation network, multi-level transfer learning, thyroid nodules, ultrasound images

## Abstract

In the past few years, deep learning has gained increasingly widespread attention and has been applied to diagnosing benign and malignant thyroid nodules. It is difficult to acquire sufficient medical images, resulting in insufficient data, which hinders the development of an efficient deep-learning model. In this paper, we developed a deep-learning-based characterization framework to differentiate malignant and benign nodules from the thyroid ultrasound images. This approach improves the recognition accuracy of the inception network by combining squeeze and excitation networks with the inception modules. We have also integrated the concept of multi-level transfer learning using breast ultrasound images as a bridge dataset. This transfer learning approach addresses the issues regarding domain differences between natural images and ultrasound images during transfer learning. This paper aimed to investigate how the entire framework could help radiologists improve diagnostic performance and avoid unnecessary fine-needle aspiration. The proposed approach based on multi-level transfer learning and improved inception blocks achieved higher precision (0.9057 for the benign class and 0.9667 for the malignant class), recall (0.9796 for the benign class and 0.8529 for malignant), and F1-score (0.9412 for benign class and 0.9062 for malignant class). It also obtained an AUC value of 0.9537, which is higher than that of the single-level transfer learning method. The experimental results show that this model can achieve satisfactory classification accuracy comparable to experienced radiologists. Using this model, we can save time and effort as well as deliver potential clinical application value.

## 1. Introduction

Nowadays, thyroid cancer is becoming more common worldwide, and the incidence rate is increasing rapidly compared with other malignant tumors. According to the American Cancer Society and cancer statistics, it is the most prevalent endocrine tumor, with 500,000 new cases identified each year (567,233 in 2018) [1]. In clinical practice, ultrasonography is the most commonly utilized test for the screening and diagnosis of thyroid gland disorders due to its non-invasive, non-radioactive nature, affordability, and real-time capabilities. However, its drawbacks include a low signal-to-noise ratio and the presence of visual image artifacts visible in the ultrasound image. In addition, diagnostic accuracy and reproducibility of ultrasound images are limited and it necessitate a high level of expertise and training [2]. In light of these challenges, many researchers have reported the importance of computer-aided diagnosing systems to characterize thyroid nodules.

Recently, there has been increased interest in the computer-aided diagnosis of thyroid nodules, and significant research progress has been made in this area. In the traditional machine learning framework, quantitative features are extracted from the ultrasound images, which are used to classify benign and malignant thyroid nodules [3]. This type of CAD system always attempts to extract morphological or texture features (such as Local Binary Patterns (LBP), Gray Level Co-occurrence Matrix) from ultrasound images [4]. However, conventional machine learning algorithms designed specifically for retrieving this type of features often require hand-crafted optimal combinations and complex processes of image preprocessing, feature extraction, identification of the best feature set and classification [4]. Another serious challenge faced by the traditional computer-aided diagnosis system is the irregular and complex appearance of the nodules in the thyroid ultrasound image, which makes feature identification and extraction difficult [4]. Moreover, the performance of any such system is greatly affected by various factors such as type of image modality, quality of the obtained image, similarity in the morphology of lesions, types of cancer, etc. [4]. A simple feature extraction method will not yield effective results and will fail to address these issues [5]. This suggests the need to use more complex convolutional neural network models to characterize thyroid nodules from ultrasound images [4].

Recently, convolutional neural networks have proven particularly effective in the analysis of radiological, pathological, and other medical image classification tasks [5]. Compared to its predecessors, the main advantage of CNN is that it automatically detects the essential features without any human supervision [5]. For example, we have many pictures of cats and dogs, and the CNN model learns the distinctive features of each class by itself. The convolutional neural network can address the above-mentioned issues associated with traditional feature extraction approaches effectively [6]. This type of approach can automatically learn deeper and more abstract features and avoid complex manual feature extraction.

Convolutional neural networks have been widely accepted as a more effective approach for identifying and evaluating the malignant nature of thyroid nodules than traditional methods. Although CNN applications work very well on large datasets, they fail to achieve significant performance on small datasets. Moreover, substantial variability in the size, shape, and appearance of the nodules in the ultrasound images hinders the development of an effective characterization model for thyroid nodule. The area occupied by the nodule and the shape of the nodule in the ultrasound image is different in each image. Consequently, a simple convolutional neural network cannot capture the essential features of the nodules in the ultrasound image. Thus, an efficient convolutional neural network architecture is necessary for the proper characterization of thyroid nodules in ultrasound images. C.Szegedy et al. proposed a deep convolutional neural network architecture (called inception) which helps to expand the width of the network and incorporate multi-scale feature information [7]. This helps to achieve efficient use of computational resources, and more significant features are extracted under the same computational requirement. Likewise, when doctors diagnose a thyroid ultrasound image, they tend to pay more attention to the places that are decisive for the diagnosis. We cannot implement this through simple CNN architecture. By incorporating the idea of attention networks for recognizing important regions by itself, it will help to improve the diagnosing capability of CAD systems. In 2018, Hu et al. proposed the concept of a squeeze and excitation network which is both efficient and more precise in recognizing complex patterns in images [8]. To achieve high efficiency, the module uses a global average pooling layer (GAP) that comparatively decreases the number of model parameters and enables the model to converge faster than existing models [9]. The squeeze and excitation network enhances the propagation of significant features and improves the prediction accuracy of the model. This paper hypothesizes that better diagnostic performance can be achieved by combining the inception architecture and the squeeze and excitation network. Also, the complex micro- and macro-structures of thyroid nodules can be extracted with the help of this approach.

Furthermore, to address issues regarding small datasets, we utilized a multi-level transfer learning mechanism using a bridge dataset from the same image modality to efficiently characterize thyroid nodules [10,11]. Here, we used a breast ultrasound image dataset of significant images as a bridge dataset. Several studies have demonstrated possible associations between breast and thyroid cancer, including shared hormonal risk factors, similarity in the appearance of nodules, and genetic susceptibility [4]. Furthermore, thyroid and breast cancers exhibit similar characteristics under high-frequency ultrasound, such as malignant nodules having a taller-than-wide shape, hypoechogenicity, and an ill-defined margin. This is why we selected the breast ultrasound image dataset as a bridge dataset for multi-level transfer learning for the classification of thyroid nodules [4].

Inspired by the above-discussed issues, this paper proposes an architecture that combines the inception architecture with the squeeze and excitation module based on the multi-level transfer learning technique for developing an efficient characterization framework for thyroid nodule diagnosis. The contributions of this paper are as follows:

- We utilize the concept of attention mechanism with each inception block and propose a network architecture for thyroid nodule diagnosis.
- We propose a multi-level transfer learning model for thyroid nodule diagnosis which uses breast ultrasound images as a bridge dataset. We utilize a new concept of multi-level transfer learning to the thyroid ultrasound images, whereas most of the previous studies are similar to ours but have remained within the traditional transfer learning technique. We test the feasibility of the model and prove its potential for thyroid nodule diagnosis.
- We check the effectiveness of breast ultrasound images for use as a bridge dataset in the development of a multi-level transfer learning model for thyroid nodule diagnosis. They are able to show the potential and usefulness in the development of a thyroid nodule classification model.

The remainder of this paper is organized as follows. Section 2 includes the related works. Section 3 discusses the proposed approach for the thyroid nodule characterization. Section 4 discusses the experimental framework. Section 5 discusses the obtained results. Lastly, Section 6 contains our concluding remarks.

## 2. Background

Thyroid nodule diagnosis using machine learning techniques has long been an important research topic that provides aid to clinical diagnosis. This section reviews the state-of-the-art approaches in the development of a computer-aided diagnosing system for thyroid nodules. In the traditional machine learning framework, several works have been proposed for the computer-aided diagnosis of thyroid nodules. In 2008, Keramidas et al. extracted fuzzy local binary patterns as noise-resistant textural features and adopted support vector machine as the classifier [12]. In 2009, Tsantis et al. proposed a model for thyroid nodule classification in which a set of morphological features (such as mean radius, radius entropy, and radius standard deviation) were extracted from the segmented nodule to describe the shape and boundary regularity of each nodule [13]. In 2012, Singh and Jindal utilized gray level co-occurrence matrix features to construct a k-nearest neighbor model for thyroid nodule classification [14]. Acharya et al. utilized Gabor transform to extract the features of thyroid ultrasound images to differentiate benign and malignant nodules. They compared the classification performance of SVM, MLP, KNN, and C4.5 classifiers. In 2014, Acharya et al. extracted grayscale features based on stationary wavelet transform and compared the performance of several common classifiers [15].

As thyroid nodules vary in shape, size, and internal characteristics, the low-level handcrafted features used in this traditional CAD method can only provide limited differentiating capacity due to their inherent simplicity and locality [16]. On the other hand, the performance of deep learning models, especially convolutional neural networks, has been superior to conventional learning methods in various visual recognition tasks. By learning hierarchical visual representations in a task-oriented manner, CNNs can capture the semantic characteristics of input images [16]. Due to this critical advantage, numerous CNN-based CAD methods have been proposed for thyroid nodule diagnosis in recent years.

In 2017, Ma et al. trained two complementary patch-based CNNs of different depths to extract both low-level and high-level features and fused their feature maps for the classification of thyroid nodules [17]. In 2017, Chi et al. utilized a pre-trained GoogleNet architecture to extract high-level semantic features for the classification of thyroid nodules [18]. Gao et al. proposed a CAD system based on multi-scale CNN model that achieved better sensitivity [19]. In 2018, Song et al. proposed a cascaded network for thyroid nodule detection and recognition based on multi-scale SSD network and spatial pyramid architecture [20]. Recently, Li et al. structured a model for the diagnosis of thyroid cancer based on ResNet50 and Darknet19 [21]. This model, despite its simplicity in structure, exhibited excellent diagnostic abilities in identifying thyroid cancer. It demonstrated the highest value for AUC, sensitivity, and accuracy compared with the other state-of-the-art deep learning models. Wang et al. conducted a large-scale study on multiple thyroid nodule classification [22]. Both InceptionResnetv2 and VGG-19 architectures were utilized for the classification [22]. It was a microscopic histopathological image(rather than an ultrasound image) was used in the investigation. Liu et al. proposed a multi-scale nodule detection approach and a clinical-knowledge-guided CNN for the detection and classification of thyroid nodules. By introducing prior clinical knowledge such as margin, shape, aspect ratio, and composition, the classification results showed an impressive sensitivity of 98.2%, specificity of 95.1%, and accuracy rate of 97% [16]. The method involves using separate CNNs to extract features within the nodule boundary, around margin areas, and from surrounding tissues [16]. As a result, the architecture of the network is complex with a higher risk of overfitting [16]. Juan Wang et al. developed an Artificial Intelligence–based diagnosis model based on both deep learning and handcrafted features based on risk factors described by ACR-TIRADS [23]. Yifei Chen et al. proposed two kinds of neural networks, which are GoogleNet and U-Net, respectively [24]. GoogleNet was utilized to obtain the preliminary diagnosis results based on the original thyroid nodules in the ultrasound images. U-Net was used to obtain the segmentation results and medical features are extracted based on the segmentation results. The mRMR feature selector was used as the feature selector. The 140 statistical and texture features were sent to the designed feature selector to obtain 20 features. Then, they were utilized for training an XgBoost classifier. The above CNN-based approaches have achieved good performance in classifications, but they still have limitations in global feature learning and modeling. CNNs always focus on the fusion of local features, owing to the locality of their convolutional kernels. Some improved extraction strategies of global features, such as downsampling and pooling, have been proposed. However, they tend to cause the loss of contextual and spatial information. Jiawei Sun et al. proposed a vision-transformer-based thyroid nodule classification model using contrast learning [25]. Using ViT helps to explore global features and provide a more accurate classification model. Geng Li et al. proposed a deep-learning-based CAD system and transformer fusing CNN network to segment the malignant thyroid nodule automatically [26].

As in the above papers, various deep learning networks, training methods, and feature extraction methods were utilized to develop an efficient thyroid nodule diagnosis model. In general, there have been many papers on applying deep learning techniques to achieve computer-aided diagnosis of thyroid nodules. However, only a few of them address the issues regarding small datasets. In 2021, Y. Chen et al. proposed a multi-view ensemble learning model based on a voting mechanism that integrates three kinds of diagnosis results obtained from thyroid ultrasound images. They utilized features from the GoogleNet architecture, medical features obtained from the U-Net architecture, and several statistical and textural features to develop the ensemble model.

To date, Artificial Intelligence–based Computer-aided Diagnosis (AI-CAD) systems have been developed for specific medical fields or specific organs. Integrating these systems and evaluating related organs with similar characteristics would benefit AI-CAD system development. For example, several researchers have reported an association between the incidence rate of thyroid and breast carcinoma, possibly related to the effect of estrogen, which is the transport mechanism of iodine. Considering that thyroid and breast nodules exhibit similar characteristics over high-frequency ultrasound, Zhu et al. developed a generic VGG-based framework to classify thyroid and breast lesions in ultrasound imaging [4]. Xiaowen Liand et al. proposed a multi-organ CAD system based on CNN for classifying thyroid and breast nodules from ultrasound images [27]. Inceptive characteristics of Googlenet and CaffeNet were exploited to classify nodules in the ultrasound images.

However, this paper mainly focuses on the concept of inception network and squeeze and excitation network. We also integrate the idea of multi-level transfer learning by considering the relationship between thyroid and breast nodule ultrasound images.

## 3. Proposed Approach

### 3.1. Framework Overview

In this paper, we mainly explore the effectiveness of parallel convolutions in the inception architecture, squeeze and excitation module and the idea of multi-level transfer learning for characterizing thyroid nodules. In Section 3.2 and Section 3.3, we introduce the basic concepts regarding the inception architecture and squeeze and excitation modules, respectively. In Section 3.4, we explained how the inception blocks are updated using the squeeze and excitation module. Section 3.5 discusses the proposed convolutional neural network based on improved inception blocks. Section 3.6 discusses the essential information regarding multi-level transfer learning and how it is implemented in the proposed system.

### 3.2. Inception Module

The most straightforward way to improve the performance of a deep neural network is to increase its size. This includes expanding the depth and the width. The depth of the network defines the number of levels of the network, and the width represents the number of units at each level [28]. It is easy to train higher-quality models, especially if a significant amount of labeled training data are available. A larger network tends to have more parameters, making it more prone to overfitting when the size of the dataset is limited [7,29]. Another drawback of a uniformly increased network size is the significantly higher demand for computational resources [7]. These issues can be resolved by switching from fully connected to sparsely connected architectures, even within the convolutions.

**Figure 1:**
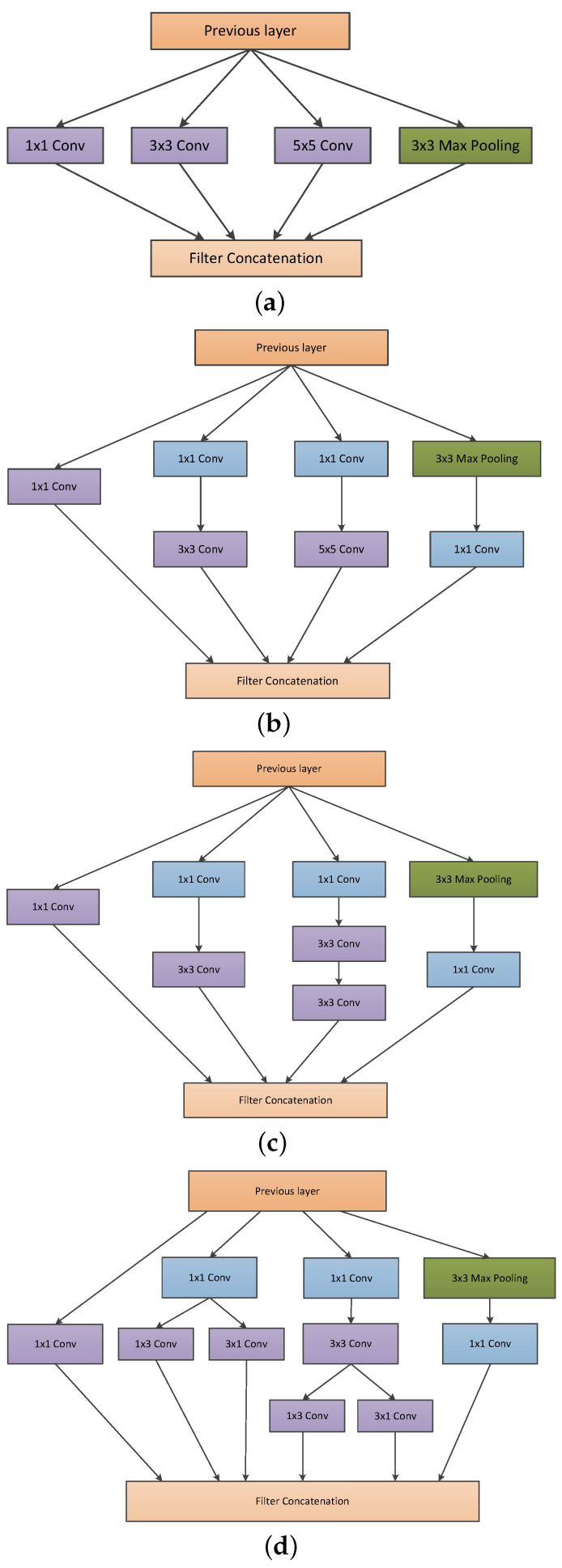
(**a**) Naive inception module. (**b**) Inception module with dimension reduction. (**c**) Factorizing the 5×5 convolution into two 3×3 convolutions. (**d**) Factorizing convolutions of filter size nxn to a combination of 1 × n and n × 1.

Likewise, the size of the significant elements of the image can vary considerably. The area occupied by one element in an image differs from that occupied by the same element in another image. Selecting the right kernel size for convolution becomes challenging due to the wide variation in the location of significant information [28]. A larger kernel is preferred for the information distributed globally, while a smaller kernel is chosen for the information distributed locally. Unlike traditional deep neural networks, inception networks are well known for parallel stacked convolutions. The inception module is illustrated by the incorporation of convolution kernels of various scales in the same convolution module [7]. As opposed to a single convolutional kernel, the inception module can extract a wide range of significant features from single-layer feature maps using a variety of convolutional kernels. It expands the dimension of each layer of the feature map without affecting neural networks as a whole [7]. Figure 1 represents various inception blocks. Figure 1a presents the naive inception module. It performs convolution on the input with three different sizes of filters: 1×1, 3×3, and 5×5, respectively. Additionally, max pooling is also performed [7]. The outputs are concatenated and sent to the next inception module.

As stated before, deep neural networks are computationally expensive. By adding an additional 1×1 convolutions before the 3×3 and 5×5 convolutions, the author reduces the number of input channels (represented in Figure 1b) [28]. Hence, it makes the network computationally cheaper. Although adding an extra operation may seem counter-intuitive, 1×1 convolutions are far more affordable than 5×5 convolutions and will reduce the number of input channels [28]. However, the 1×1 convolution is introduced after the max pooling layer. In [7], a new neural network architecture was developed using the improved inception module. It was referred to as GoogLeNet (Inception-v1). GoogleNet consists of nine linearly stacked inception modules. It is 22 layers in depth (27 layers deep, including the pooling layers). At the end of the last inception module, a global average pooling is applied. The concept of auxiliary classifiers is introduced to prevent the middle part of the network from “dying out”. SoftMax is applied to the outputs of two of the inception modules. Moreover, an auxiliary loss is computed over the same labels [28]. The weighted sum of the auxiliary loss and the real loss is calculated, and it is considered as the total loss [28]. For each auxiliary loss, the weight value is fixed as 0.3 [28].

(1) $$total_{loss}=real_{loss}+0.3*aux_{loss_{1}}+0.3*aux_{loss_{2}}$$

**Figure 2:**
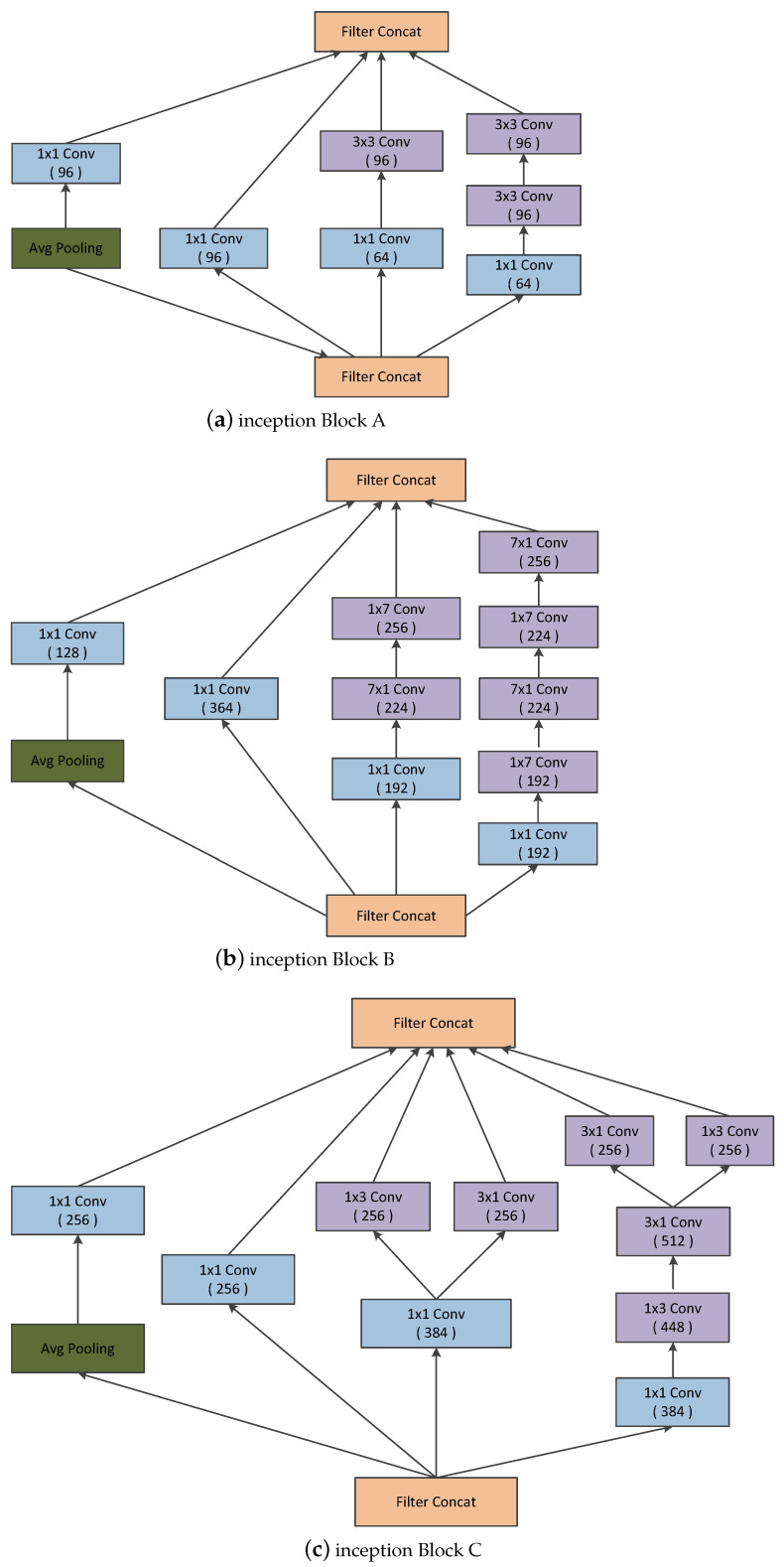
Various inception modules.

Two versions of inception have been presented together in a single paper: Inception-v2 and Inception-v3 [28]. The authors proposed a number of upgrades which increased the accuracy and reduced the computational complexity. Inception-v2 explores the following:

- Reducing the Representational Bottle Neck: The neural network performs better when convolutions did not alter the dimensions of the input drastically. Reducing the dimensions too much may cause loss of information, known as representational bottle neck.
- Convolution operations can be made more efficient in terms of computational complexity by using some smart factorization methods.
  For instance, factorize the 5×5 convolution to two 3×3 convolutions to improve computational speed (represented in Figure 2a). Although this may seem counter-intuitive, a 5×5 convolution is 2.78 times more expensive than a 3×3 convolution. Hence, stacking two 3×3 convolutions in fact leads to a boost in performance. This is illustrated in Figure 2a. Moreover, they factorize convolutions of filter size n×n to a combination of 1×n and n×1 convolutions) [30]. For example, a 3×3 convolution is equivalent to first performing a 1×3 convolution and then performing a 3×1 convolution on its output (represented in Figure 2b. They found this method to be 33% cheaper than the single 3×3 convolution.
- In addition, the filter banks in the module were expanded (made wider instead of deeper) to address the issue of representational bottleneck. In contrast, if the module were made deeper, the dimensions would be reduced excessively, resulting in information loss. This is depicted in the Figure 2c.

The above three principles were used to develop three different types of inception modules (Let us call them modules A, B, and C in the order in which they were introduced). Inception-v3 incorporated all of the above upgrades stated for Inception-v2, and in addition used the following [28]:

- RMSProp Optimizer [28].
- Factorized 7×7 convolutions [28].
- BatchNorm in the fully connected layer of Auxillary Classifiers: The authors observed that the auxiliary classifiers did not make a significant contribution until near the end of the training phase, when accuracies were approaching saturation. In particular, they claimed that they act as regularizers, especially if they had BatchNormalization or Dropout operations [28].
- Label Smoothing: It is a regularization technique that can address issues regarding overconfidence and overfitting behavior of a convolutional neural network [28,31].

**Figure 3:**
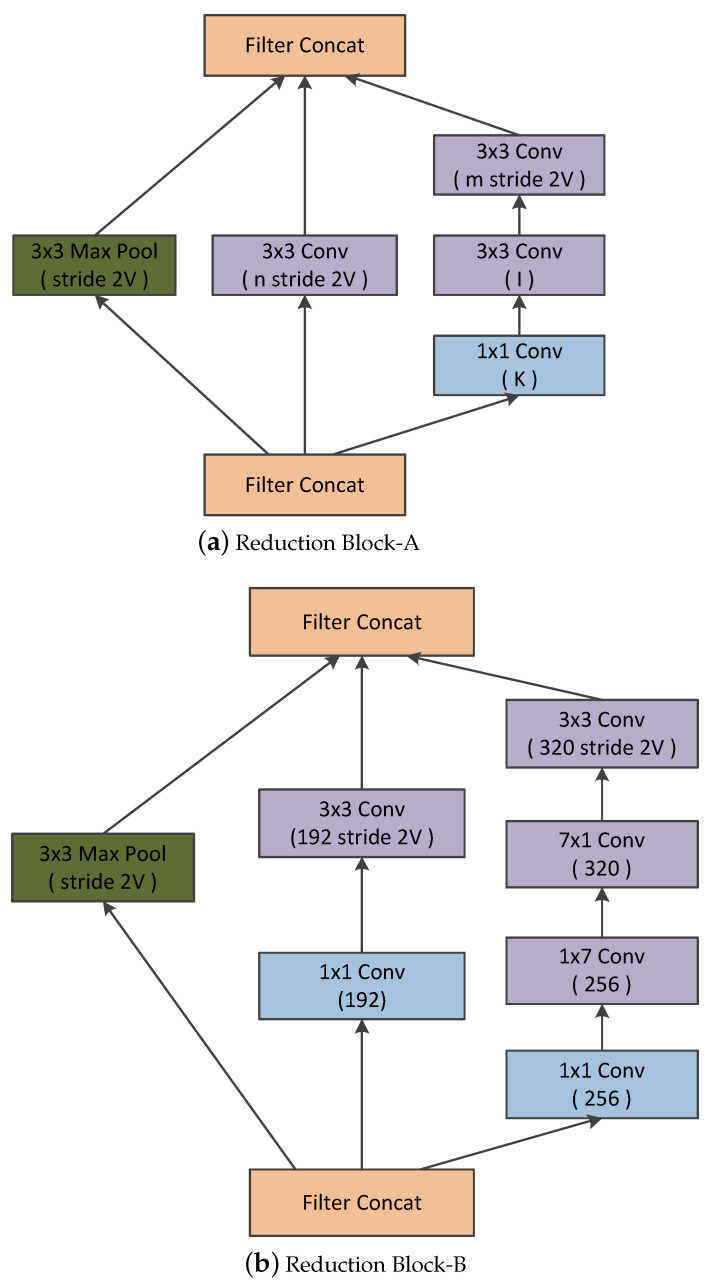
Various Reduction Modules.

Inception-v4 was introduced by Christian Szegedy et al. in 2016 [32]. It had three main inception modules, which are termed A, B, and C, which are very similar to those from Inception-v2 (or v3). Inception-v4 introduced a specialized reduction block that changes the width and height of the grid (represented in Figure 3). Even though the functionality of the reduction blocks was incorporated in the earlier version, they were not explicitly implemented [33].

### 3.3. Attention Mechanism and Squeeze and Excitation Networks

Recently, attention mechanisms have been widely used in pattern recognition and have been proven effective. In contrast to natural images, medical images tend to be similar in appearance. Even though they are from different image sources, they are acquired from standardized positions using similar set of acquisition parameters. For radiologists, experience in analyzing the images is associated with knowing where exactly to look to detect specific abnormalities in the images [8]. Furthermore, extensive variability in the shape and appearance of nodules in the ultrasound images led to false positive predictions [34]. To address these issues, for reducing false positive predictions, we used squeeze and excitation networks because they used fewer parameters and provided superior results when compared with other techniques. In fact, we claim that the inability to exploit global information is a common problem in medical image analysis. This type of network is a modular mechanism that allows for the efficient exploitation of global information, which also provides soft object localization during forward pass. This type of network helps to focus on regions that are disease-specific. Generally, this strategy is particularly effective for focusing on nodule regions. It can reduce the impact of the noise in the non-nodule region, and misalignment can be alleviated. In the attention mechanism, re-weighting of certain features of the network has been accomplished with the help of some externally or internally (self attention) supplied weights [9]. In order to understand what a model is doing from an attention point of view, we need to familiarize with both hard attention and soft attention. Soft attention allows their weights to be continuous, while hard attention requires them to be binary (0 or 1) [8]. In the case of hard attention, certain parts of the image are cropped out. In essence, the original image is re-weighted so that the cropped part has a weight of 1 and the rest has a weight of 0. It is not differentiable and cannot be trained end-to-end, which is the main disadvantage of hard attention. Instead, the authors use the activation of a certain layer to determine the ROI and train the network in a complicated multistage process [9]. In order to train the attention gates, we have to use soft attention. Instead of using hard attention and recalibrating weights in terms of cropping the feature maps, Hu et al. looked at re-weighting the channel-wise responses in a certain layer of a CNN by using soft self attention to model interdependencies between the channels of the convolutional features [9]. For this purpose, the authors proposed the concept of squeeze and excitation building block. Normally, the network weights each of its channels equally when creating the output feature maps. The squeeze and excitation Networks (SENets) are all about changing this by adding a content-aware mechanism to weight each channel adaptively. It adds a single parameter to each channel and gives it a linear scale of how relevant each one is. First, they obtain a global understanding of each channel by squeezing the feature maps to a single numeric value. This results in a vector of size n, where n is the number of convolution channels. Afterwards, it is fed through a two-layer neural network, which outputs a vector of the same size. These n values can now be used as weights on the original feature maps, scaling each channel based on importance.

**Figure 4:**
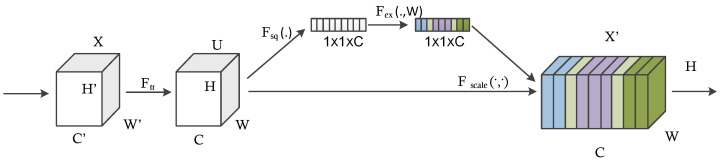
Squeeze and Excitation Block.

The squeeze and excitation block work as follows. For any transformation of features $F_{tr}$ from X to U (e.g., Convolution), there is a transformation $F_{sq}$ that aggregates the global feature responses across spatial extents (H,W) [9]. This represents the squeeze operation. The squeeze operation is followed by the excitation operation $F_{ex}$, which is a self-gating operation that constructs a channel-wise weight response [8]. The output of $F_{tr}$ is subsequently multiplied channel-wise by the result of excitation. This is depicted as $F_{scale}$ in Figure 4 [35].

The mathematical representation of the squeeze operation is as follows [9].

(2) $$z_{c}=F_{sq}\left(u_{c}\right)=\frac{1}{H\times W}\sum_{i=1}^{H}\sum_{j=1}^{W}u_{c}\left(i,j\right)$$

(3) $$s=F_{ex}\left(z,W\right)=\sigma \left(g\left(z,W\right)\right)=\sigma \left(W_{2}\delta \left(W_{1}z\right)\right)$$

Here, $u_{c}$ are the outputs of the operation $F_{tr}$. The squeeze operation uses global average pooling to create a global embedding. We could also use global max pooling. The authors note that average pooling slightly improves the overall performance [35]. The excitation block is represented by:

(4) $$s=F_{ex}\left(z,W\right)=\sigma \left(g\left(z,W\right)\right)=\sigma \left(W_{2}\delta \left(W_{1}z\right)\right)$$

The significant advantage of the squeeze and excitation block is in incorporating global information while decision making [8]. Conversely, a convolution operation focuses on local spatial information within a specific area [9]. According to the authors, in the early stages of the network, excitation weights are almost the same for different classes but become more specific in later stages [9]. In other words, lower layers of the network learn more general input features, whereas higher layers are more likely to be specific [9]. Additionally, the squeeze and excitation block does not make much sense at the last stage of the network, where most excitations become one [35]. This can be explained by the fact that the last stage of the network already contains most of the global information and the squeeze and excitation operation brings in no new information content [35]. squeeze and excitation building blocks offers the advantage of being extremely versatile; the authors mention that it can integrate with any convolutional neural network architecture, such as ResNet and Inception. We can integrate the squeeze and excitation building block to every stage of the network or just at certain stages. Additionally, it introduces only a slight overhead concerning the number of learnable parameters.

**Figure 5:**
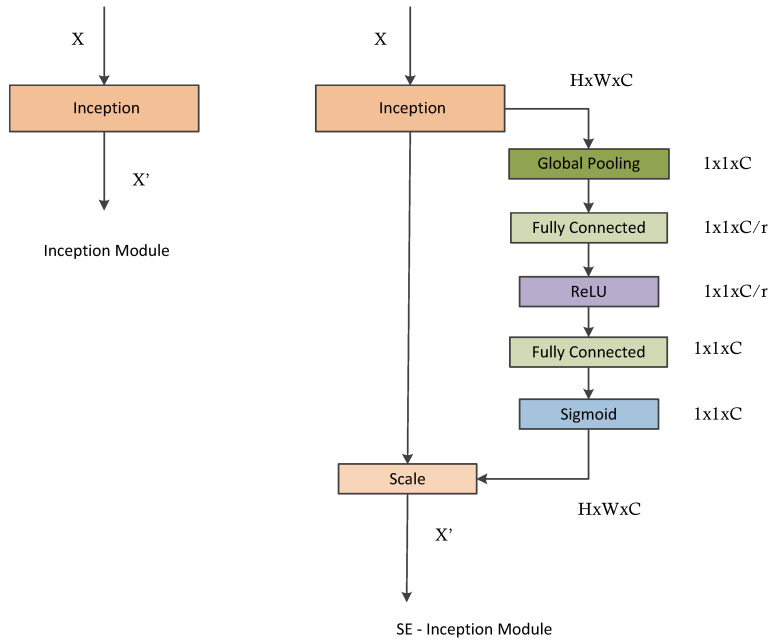
The schema of the original inception module (**left**) and the SE-inception module (**right**).

Therefore, we consider the construction of SE-blocks for inception modules. Here, we simply transform $F_{tr}$ to be an entire inception module (see Figure 5). By making this change for each such module (see Figure 2) in the architecture, we obtain an SE-Inception network [9]. This will help the network to learn the importance of channel in the process of network training.

### 3.4. Proposed Improved Inception Squeeze–Excitation Blocks

Jie Hu et al. proposed an inception architecture combined with the squeeze and excitation module, which is given in Figure 5. In this architecture, the SE-block is inserted into every inception block. Assessment of ultrasound images for thyroid nodule diagnosis is based on the experience of the clinicians. On ultrasound images, clinicians tend to focus on certain places pertinent to diagnosis when examining thyroid nodules. We include the SE-block in each inception module to ensure that the inception modules learn these key areas independently during the network training process. Accordingly, the SE-block produces corresponding attention heat maps of the same size as the traditional inception blocks. We define that all the values have a range of (0, 1) activated by the sigmoid activation function. If the value of the heat map is closer to 1, the more the location is concerned by the network. Finally, the significant feature map is obtained by multiplying the attention heat map by the corresponding feature map produced by the traditional inception block. In other words, the more focused on the pixel, the more completely retained the feature value and vice versa. Thus, in the process of gradient descent during network training, when the classification is correct, the weight of the feature will be increased and vice versa. Eventually, the network reduces the attention values in the irrelevant part and learns the significant features for classification.

**Figure 6:**
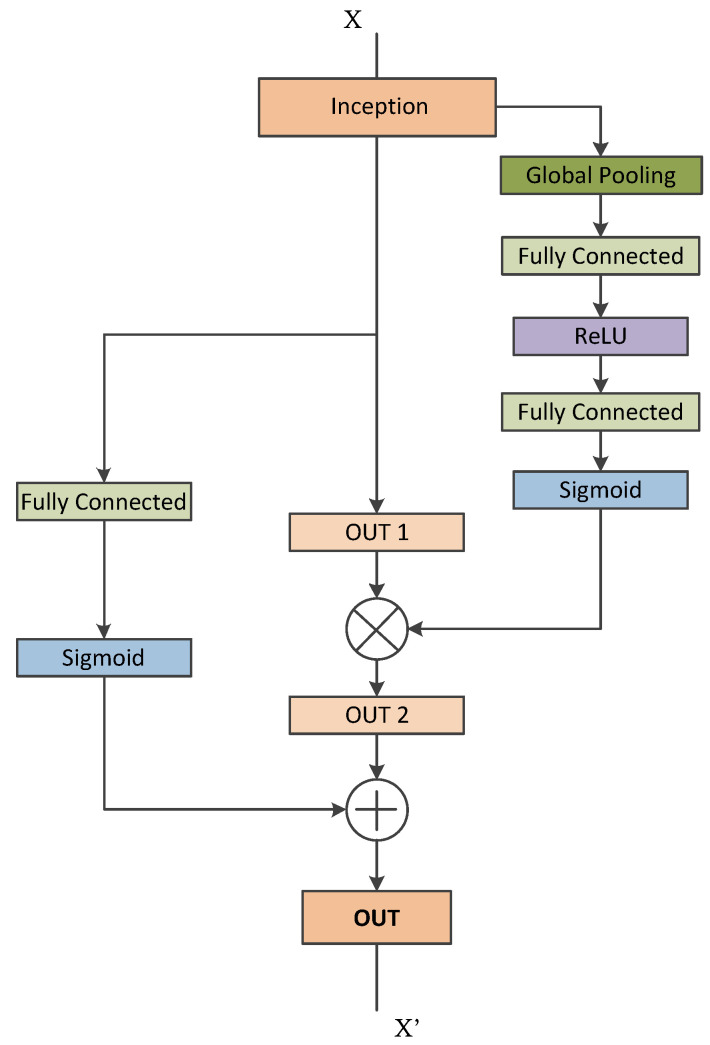
Improved inception attention module.

Furthermore, the inception module is distinguished by incorporating convolutional kernels of different scales within the single convolutional module. The advantages are obvious: Instead of using a single convolutional kernel, the inception module can extract multiple types of features from a single layer feature map using multiple convolutional kernels that expand the dimension of each layer of feature maps without increasing the depth of the network. We believe that this module brings a new problem as well: the feature map dimension disaster [36]. Feature maps of thousands of channel appear in the final concatenation layer (As shown in Figure 2). However, these channels are not fully utilized, as doctors could hardly consider so many channels when examining thyroid ultrasound images [36]. Therefore, we introduce a fully connected module along with the squeeze and excitation block and inception module [36]. The improved inception blocks along with the SE-blocks are given in Figure 6.

The improved inception blocks allow the network to learn the significant features that are crucial for the diagnosis, fully utilizing the features obtained from parallel convolutions. Figure 6 presents the improved version of the different inception blocks used in the traditional inception architecture [30,33,36].

### 3.5. Proposed Network

**Figure 7:**
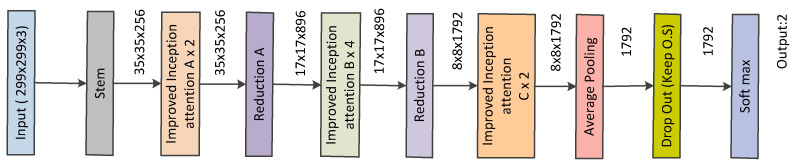
Network Architecture.

**Figure 8:**
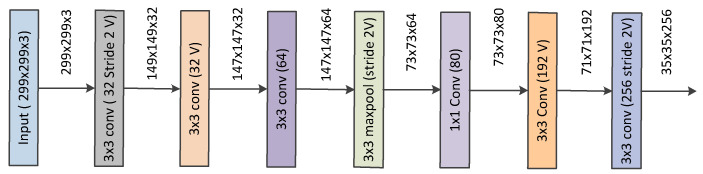
Stem of the network.

The proposed network architecture is based on Inception-v4 and it is shown in Figure 7. In this work, the stem of Inception-v4 was kept similar. However, the inception modules were updated to obtain a more lightweight network (please refer to Figure 2, Figure 3, Figure 6, Figure 7 and Figure 8). The stem refers to the initial set of operations performed before introducing the inception blocks (please refer to Figure 8). Figure 7 presents the complete architecture of the network and Figure 2, Figure 3, Figure 5, Figure 6 and Figure 8 show the detailed structure of its components. Our proposed architecture contains improved inception blocks that utilize parallel stacked convolutions and attention mechanism, and it is shallower and narrower. The number of layers and number of filters are reduced, compared with the conventional Inception-v4. This was applied to reduce computational cost and, at the same time, produce a smaller model with less capacity which would be less prone to overfitting. Unlike in Inception-v4, we have not utilized an auxiliary classifier, since our network is not as deep. The Relu activation was used after every convolution due to its simplicity and efficacy. All the convolutions marked with V are valid padded, which indicates that the input patch of each unit is fully contained in the previous layer and the grid size of the output activation map is reduced accordingly. All the convolutions that are not marked with V are same padded, which indicates that the their output grid matches the size of their input. The default input size for the proposed Inception network is 299×299. Thus, we resized the ultrasound images in a dataset into this size.

### 3.6. Overall Framework of the Multi-Level Transfer Learning for Thyroid Nodule Classification Using Breast Ultrasound Images

Recently, deep learning has taken over the field of image processing with its state-of-the-art performance. The problem is that deep learning models require enormous amounts of well-labeled training data. Generally, medical image datasets are difficult to access due to the rarity of the diseases and to ethical issues. In addition, manually collecting a massive amount of high-quality, labeled medical images is a time-consuming, labor-intensive, and expensive process. Therefore, insufficient training data makes it challenging to build an appropriate deep learning model [37]. These are the main challenges faced while developing a deep-learning model for thyroid ultrasound images. Thus, transfer learning has been introduced to address these concerns in the domain. Transfer learning involves transferring knowledge between different domains and tasks to create robust and flexible target models [38]. Transfer learning consists of using models trained for specific tasks and leveraging the model they acquired on different but related tasks. This can be highly advantageous when sufficient instances are not available for direct training on the target domain. In addition, traditional deep learning models require training from scratch, which is computationally expensive and requires a large amount of data to achieve high performance. The transfer learning approach is computationally efficient and helps to achieve better results using small datasets. Transfer learning achieves optimal performance faster than the traditional machine learning model. The model that leverages knowledge from previously trained models already understands the basic features. The traditional machine learning model has an isolated training approach where each model is independently trained for a specific purpose without dependency on past knowledge. Contrary to that, transfer learning uses knowledge acquired from a pre-trained model is applied to the development stage of the target model.

Transfer learning algorithms generally assume that the source and target domains share some information. Many real-world applications, like medical image processing and recommendation systems, do not always conform to this assumption. Moreover, knowledge transfer between two loosely related domains usually causes a negative transfer, meaning that the knowledge transfer adversely affects the performance of the task in the target domain, producing worse performance than the traditional deep learning model [37]. Medical and natural images vary significantly in several aspects, such as shape, color, resolution, and dimensionality. Compared with medical images, natural images appear diverse and possess more contour details and colors reflecting rich visual information. By contrast, medical images look almost identical, indicating considerably lesser visual information. Accordingly, natural image tasks are usually accomplished by identifying major morphological characteristics such as edges, colors, and shapes of the objects. In contrast, in medical image applications, pathologies are identified by detecting small abnormalities and local texture variations, such as bleeding and inconsistent structures. For instance, the signal-to-noise ratio of natural images is exceptionally high compared to that of medical images. Specifically, natural images have virtually no noise and are usually high-contrast and high-resolution images. Meanwhile, medical images are often noisy, and low contrast and low spatial resolution can limit detection of medical components in the images(such as nodules, cysts, bloodflow). It is obvious that such differences can hinder the effective transition of learned features from natural images for analyzing medical images. These modality differences can significantly undermine transfer learning performance. Moreover, there can be considerable modality differences even between different medical images (MRI and CT) [39]. However, the modality difference between different medical images is not significant when compared with the modality difference between medical and natural images. In addition, it was recently proven that the performance of the pre-trained model declined when they were employed for images such as chest X-ray, ultrasound, and brain MRI [40].

In 2021, J.C Hung and J.W Chang proposed the concept of multi-level transfer learning to address the issues regarding modality differences that exist among different domains [41,42]. In this approach, if optimal performance is gained at one level, knowledge gained by the corresponding model will be transferred from the current level to the next higher level.

**Figure 9:**
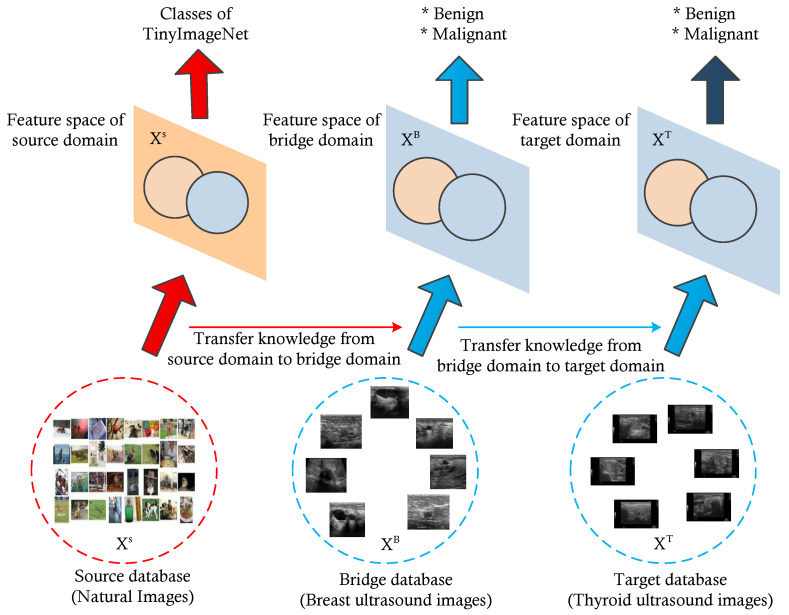
Overall framework for multi-level transfer learning for the classification of thyroid nodules from ultrasound images.

In 2017, Kim et al. proposed an approach, named modality bridge transfer learning, to address issues regarding insufficient data in the medical domain. Here, a bridge domain is introduced between the source and target domain to address the modality gap between the source and target domain. Figure 9 shows the overall framework of the modality bridge transfer learning proposed by H.G Kim et al. [39]. In a single level transfer learning approach (or traditional transfer learning), to extract image characteristics such as edge and texture, we learn the projection function,which is mapping from source image space to source feature space through the source dataset. The source database consists of a large number of natural images. To learn the features of medical images, the knowledge learned from the source domain is transferred to the target domain. However, this model does not reflect the characteristics of the target domain due to the domain difference. To learn the characteristics of the target domain (medical image), the model obtained from the source domain is supposed to be fine-tuned with images of target domain. As mentioned earlier, in the medical imaging domain, it is difficult to collect a large number of labeled images because of patient privacy protection and the high cost of reliable labeling. For this reason, training the model using this small dataset may cause overfitting or failure to converge during training. Therefore, in modality bridge transfer learning, a bridge domain is introduced between the source and target domain. A bridge domain will be from the same modality or almost similar modality to that of the target domain, but it is better in terms of the number of training samples. By learning the model from the source to the bridge domain and then from the bridge to the target domain, the domain differences between the source and target domain can be reduced. This provides a two-level approach for transfer learning. The knowledge gained from natural images (basic image features) is transferred to the bridge domain to learn the abstract features of the bridge domain. Finally, based on the learned features from two domains (source domain and bridge domain), the model is fine-tuned with the images in the target domain [43]. This particular model is applied for the different tasks in the target domain.

Based on these concepts, we utilized a multi-level transfer learning approach for the thyroid nodule classification problem, which consists of three domains: the source domain (natural images), bridge domain (breast ultrasound images), and target domain (thyroid ultrasound images) [44]. Here, we selected breast ultrasound images as the bridge domain due to the similar characteristics of thyroid and breast nodules. The bridge and target domain both belong to ultrasound images (breast ultrasound images and thyroid ultrasound images).

## 4. Experimental Framework

### 4.1. Datasets

For applying the transfer learning technique to the thyroid ultrasound image classification model, the proposed network was evaluated using four different benchmark datasets: a part of CIFAR-10 [45], a part of CIFAR 100 [45], and a part of TinyImageNet [46]. Based on the performance of the network on these datasets, we selected the part of TinyImageNet [46]. The TinyImageNet dataset contains square images of 64×64 pixels. There are three channels for color in almost all images, meaning they are 64×64×3 arrays. However, 18% of the examples are grayscale images [46]. The images are instantly converted from grayscale to RGB by replicating pixel values across three channels [46]. Each image belongs to exactly one of the 200 categories. The entire training set for TinyImageNet consists of 100K images (500 images from each category). The validation and test set has 10K images each (50 from each category) [47]. Due to the high computational requirements, we selected a subset from the training set consisting of 20K images (100 from each category). Likewise, the test set contains 2K images (10 images from each category). Another advantage of choosing TinyImageNet is the presence of grayscale images. All the images in the bridge domain (breast ultrasound) and the target domain (thyroid ultrasound) are grayscale.

For the multi-level transfer learning, we utilized a breast ultrasound image dataset as the bridge dataset [44]. This dataset consists of 1312 images, where 891 images exhibit benign nature and 421 images exhibit malignant nature. At this stage, we utilized pre-trained weights taken from the model trained on the part of TinyImageNet [46]. We selected this bridge dataset based on the approach proposed by Y-C Zhu et al. [4]. They proposed a generic deep-learning algorithm to classify thyroid and breast lesions [4]. Both breast and thyroid nodules are similar in basic internal or external characteristics and hormonal influences. Several studies demonstrated that a high frequency of thyroid-stimulating hormones and estrogen might contribute to the pathological evaluation of breast nodules and thyroid nodules [48,49]. Possible correlations between breast and thyroid cancer have also been explained in [4], as well as hormonal risk factors and genetic susceptibility. Under high-frequency ultrasound scans for malignant lesions, thyroid and breast nodules show similar imaging characteristics, including being taller than wide, having hypoechogenicity, and having ill-defined margins [4]. This observation strongly motivates using the breast ultrasound image dataset as the bridge dataset in multi-level transfer learning to classify thyroid nodule ultrasound images.

**Figure 10:**
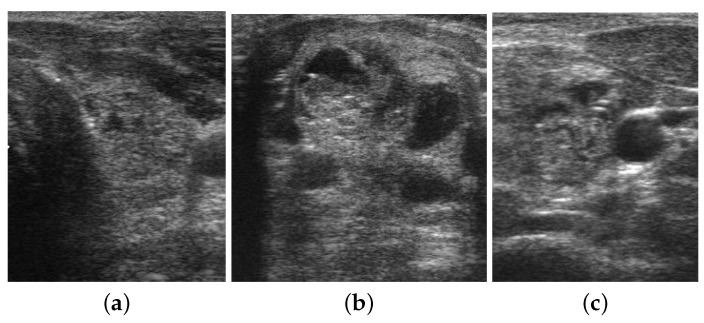
Sample images from the DDTI dataset. The first image (**a**) and the last image (**c**) represent benign nodules and the middle image (**b**) represents a malignant nodule.

For the thyroid nodule dataset, we initialized the proposed network with pre-trained weights taken from the breast ultrasound image classification model. We utilized an open-source thyroid nodule image dataset named DDTI. It contains ultrasound nodular thyroid images. Currently, DDTI contains 980 ultrasound images in total (322 images exhibit malignant nature and 658 images exhibit benign nature), around 60% for the training set and around 40% for the validation and test sets. This dataset was collected and published by Pedrazza et al. in 2015 [50,51,52]. The proposed database includes B-mode ultrasound images with a complete annotation and diagnostic description of suspicious thyroid lesions by expert radiologists [53]. The dataset includes several types of lesions like thyroiditis, cystic, adenomas, and carcinomas, and accurate lesion delineation is provided in an XML format. The diagnostic confirmation of malignant lesions was confirmed by their histopathological analysis [53]. Some sample images from DDTI dataset are given in Figure 10.

The details of the breast and thyroid ultrasound image dataset are given in Table 1.

### 4.2. Implementation Details

Our experiments were conducted on a PC with the following specifications: Intel (R) Core (TM) i7 7700 HQ with 16 GB RAM clock speed or frequency of CPU @ 2.80 GHz and a GPU of NVIDIA GeForce GTX 1080 Ti. The algorithms were implemented in Python 2.7 using the Anaconda 64-bit Windows platform. The OpenCV, Skearn, Keras, and TensorFlow libraries were used to develop the machine learning model.

### 4.3. Training Setting and Hyperparameter Setting

In the training phase, the initial value of the learning rate ($init_{lr}$) was set to 0.001 and it was attenuated according to the formula.

$$new_{lr}=init_{lr}\times \gamma^{\frac{epoch}{stepsize}}$$

where γ was set to 0.5 and $step_{size}$ was set to 4. For adequate training, we empirically set the epoch to 100, as most training procedures converge around it. We randomly split the dataset 85:15 at the patient level to create independent training and test sets. The training data was further split 90:10 to create an independent validation set. The splits were carried out in a stratified fashion to maintain the same proportion of cancer cases in the training, validation, and test sets. For the breast ultrasound dataset (bridge domain), the total number of images in the training, validation, and testing were: 1004, 111, and 197, respectively. For the DDTI dataset (target domain), the total number of images in the training, validation, and testing sets were: 750, 83, and 147, respectively.

We used TensorFlow 2.0 with Keras API to train, evaluate, and predict all the models. All the hyperparameters for the three stages of model training are given in Table 2.

Initially, for the source dataset, the network was trained using a mini-batch stochastic gradient descent algorithm. Binary cross-entropy was used as a loss function. As we already said, the learning rate was set at 0.001. The number of training epochs was set as 100 with an early stop mechanism, which would cease the optimization process if 20 consecutive epochs returned the same validation loss errors. Additional details of the model training are provided in Table 2. In the case of the bridge domain, RMSProp was used as an optimizer and binary cross-entropy as a loss function. Here, we utilized a slower learning rate to avoid overfitting. For the target domain, adam was used as the objective function and binary cross-entropy was used as the loss function. For the target domain, we followed similar hyperparameters as the bridge domain.

Overfitting may adversely affect the performance of the model when it deals with previously unseen data. Dropout methods, which temporarily remove specific nodes from the model and reduce its complexity, can help to avoid this problem. Expanding the training set could eliminate the overfitting issue in the training process. Hence, we utilized a data augmentation strategy to expand the training set. By using the ImageDataCreator package from the Keras library, we generated batches of tensor images with real-time data augmentation. All the parameters, along with the values, are shown in Table 3.

### 4.4. Data Preprocessing

The grayscale thyroid ultrasound image is 380×580 in the dataset. We modified the input to 299×299 to overcome the effects of image distortion.

### 4.5. Evaluation Metrics

In this study, we computed the accuracy, precision, recall, f1-score, g-mean and specificity of each class. Likewise, we computed accuracy, specificity, sensitivity, f1-score, and g-mean for the entire model for thyroid nodule classification. They are defined as:

(5) $$Accuracy=\frac{TP+TN}{TP+TN+FP+FN}$$

(6) $$Precision=\frac{TP}{TP+FP}$$

(7) $$Recall=\frac{TP}{TP+FN}$$

(8) $$f1-score=\frac{2\times Precision\times Recall}{Precision+Recall}$$

(9) $$Specificity=\frac{TN}{TN+FP}$$

The performance of the model was also evaluated using the Receiver Operating Characteristics Curve (ROC Curve).

## 5. Results and Discussions

In this section, the experimental results are discussed to validate the performance of the proposed model for the characterization of thyroid nodules. Section 5.1 discusses the performance of the obtained intermediate models based on both the selected part of TinyImageNet and the breast ultrasound images. Section 5.2 deals with interpreting the training and validation curves of classification models obtained for thyroid ultrasound images. Section 5.3 summarizes the results of evaluating various models based on a test set in terms of several evaluation matrices. Section 5.5 interprets the receiver operating characteristic curves associated with each implemented model. Section 5.6 illustrates the results of several state-of-the-art methods for thyroid nodule characterization. Section 5.7 explains several benefits of the proposed method, and Section 5.8 discusses several limitations of the current study and some future directions from it.

### 5.1. Evaluation of the Proposed Network for Breast Cancer Ultrasound Images

First, the network is trained with part of TinyImageNet, as discussed in Section 4.1. Then the bridge dataset, the breast ultrasound image dataset, acts as a bridge across the source and target domains by constructing a high-level feature space and reducing the corresponding distribution divergences. The performance of the network for the source domain (part of TinyImageNet) is reported in Table 4 as Phase 1. It achieves an accuracy of 0.9857, a precision of 0.9790, a recall of 0.9286, and an F1-score of 0.8975. In Phase 2, the network trained using the part of TinyImageNet is finetuned using the breast ultrasound image dataset and achieves an accuracy of 0.8967, precision of 0.8567, recall of 0.9286, and an F1-score of 0.8340. The performance of the network for these classifications is much better. Hence, it is suitable for transfer learning. Next, we try to transfer the network parameters to the target domain to characterize thyroid nodules in ultrasound images.

### 5.2. Evaluation of the Proposed Method

**Figure 11:**
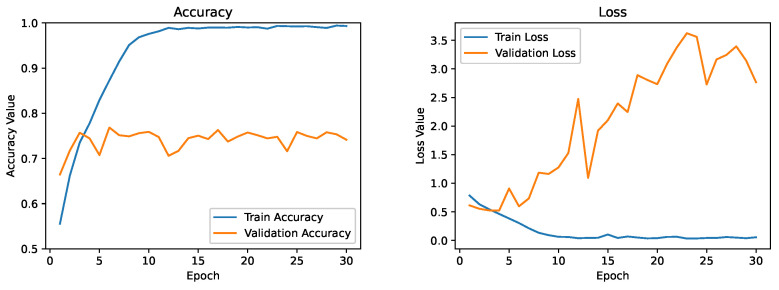
Training and validation curve for the baseline model.

As a first attempt, the architecture proposed in Section 3.5 was trained from scratch using the DDTI dataset. In the remainder of the article, this model will be referred to as the baseline model. Figure 11 depicts the evolution of the running average of the training and validation accuracy and loss function. However, we noticed that the model severely overfitted the training data at 30 epochs. The training accuracy had already exceeded 90% and was improving rapidly. At the same time, the validation accuracy remained stable at around 75%. We can see a significant deviation in training loss and validation loss. It suggests that applying more regularization approaches to our model could help it generalize to validation sets or data that have never been seen before.

**Figure 12:**
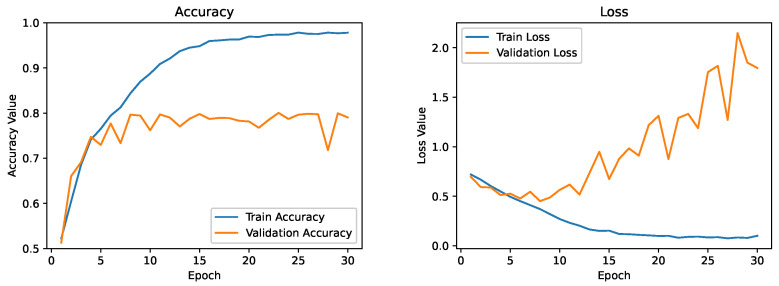
Training and validation curve for the model with regularization.

Next, we improved our baseline model by adding more convolutional and dense layers. We add a dropout of 0.3 after each hidden dense layer to facilitate regularization. Dropout is a powerful regularization method for deep convolutional neural networks. It can be applied separately to both the input and hidden layers. The dropout layer sets the output of a few layers to zero to prevent overfitting (in our case, 30% of the units in dense layers). Figure 12 depicts the training and validation curve for the regularized model. It is evident from the training and validation curves that the model still continues in a state of overfitting. However, it takes slightly longer, and our validation accuracy is somewhat better, which is decent but not amazing. Due to limited training data, the model continuously sees the same occurrences across epochs, leading to model overfitting. The solution to this challenge would be to augment images in our training set using an image augmentation strategy that uses minor alterations to existing data.

**Figure 13:**
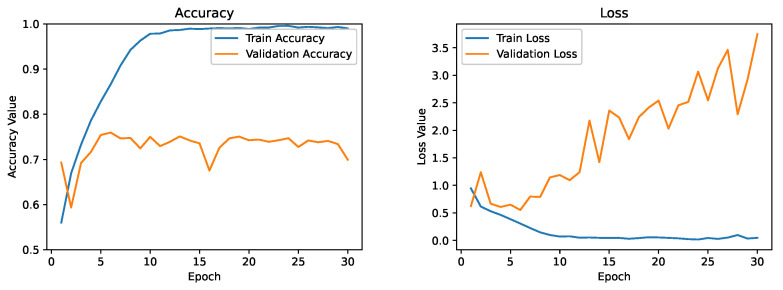
Training and validation curve for the regularized model + data augmentation.

For the next attempt, we added data augmentation at training time. This means that we added a preprocessing step to each batch before training. Each image was randomly flipped from left to right and altered in brightness and contrast. All the details of data augmentation are given in Section 4.3. The training parameters described in the earlier attempts were kept identical. The validation and training accuracy are plotted and given in Figure 13. While there are some spikes in the validation accuracy and loss curves, we can see a significant improvement in the validation accuracy. It is significantly closer to training accuracy, which indicates the generalization capability of the model compared to our previously obtained models. Here also, the variation in training and validation accuracy curve indicates the state of model overfitting.

**Figure 14:**
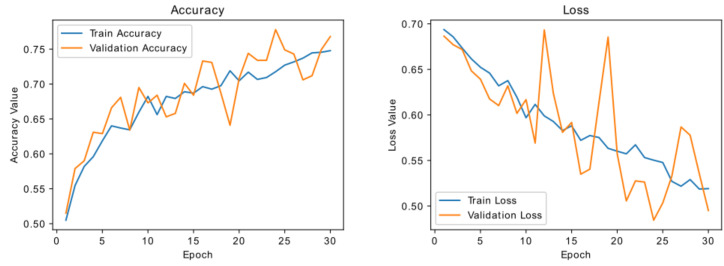
Training and validation curve for the model which uses transfer learning from TinyImageNet.

A way to combat this would be to adopt transfer learning strategies. Therefore, we trained the model using a massive dataset with a more significant number of instances (here, we used a subset of TinyImageNet). During this stage, the network can learn a robust hierarchy of features: spatial, rotational, and translational invariants. The network can extract relevant features from the images using this pre-trained model for thyroid nodule classification. Here, the network is fine-tuned with thyroid ultrasound images from DDTI datasets. As a result, we achieved a validation accuracy of close to 76%, which is an improvement of almost 6–7% from our basic CNN model with image augmentation. The model does seem to be overfitting, though. After the fifth epoch, there is a substantial gap between model training and validation accuracy curves, suggesting that the model is in the state of overfitting. As of now, however, this appears to be the best model. The training and validation curves for the model are shown in Figure 14.

**Figure 15:**
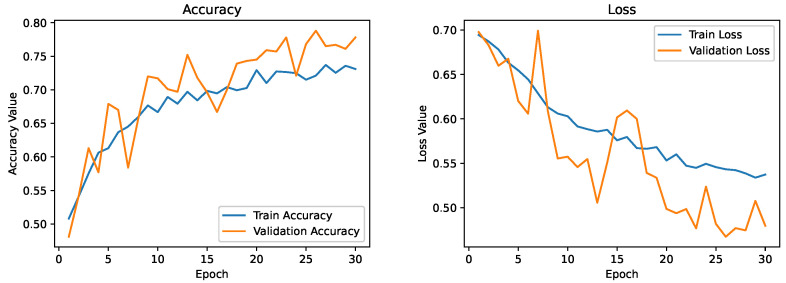
Training and validation curve for the model which uses transfer learning based on TinyImageNet and breast ultrasound images.

As a next step, we tried multi-level transfer learning based on TinyImageNet and the breast ultrasound images. We can see the improvement in training and validation accuracy and the corresponding loss. As a result, we obtained a better classification model with a validation accuracy of 80%, which is nearly a 5–6% improvement over our previous CNN model which used single-level transfer learning. The training and validation curve for the model is shown in Figure 15.

### 5.3. Evaluation Metrics

We evaluated the performance of all the models, starting with the baseline model, in terms of different evaluation metrics. Table 5, Table 6 and Table 7 shows the performance of all the models, starting with the Data Augmentation + Regularization model. The results obtained for all the models (data augmentation + regularization, single-level transfer learning, and multi-level transfer learning) are given in Table 5, Table 6 and Table 7. We also included different characterization models developed from different pre-trained CNN architectures, and the performance of each model is given in Table 8. The performance of each class (for Inception-v3, Inception-ResNet v2) is shown in Table 5. Table 5 lists the precision, recall, and F1-score results for both benign and malignant classes. Table 6 lists the G-Mean and specificity for both benign and malignant classes.

As presented in Table 7, the precision of the first model (Data Augmentation + Regularization) without transfer learning was 88.68% for the benign class and 93.30% for the malignant class, and training was performed using a single level transfer learning and multi-level transfer learning to improve the accuracy further. In the case of single-level transfer learning, it achieved a precision of 90% for the benign class and 93.33% for the malignant class. In the case of multi-level transfer learning, it obtained a precision of 0.9057 for the benign class and 0.9667 for the malignant class. Both approaches based on transfer learning achieved higher precision when compared with the basic CNN approach.

In the case of recall, the recall for the proposed approach based on multi-level transfer learning is 0.9796 for the benign class and 0.8529 for the malignant class, while that of single-level transfer learning is 0.9600 for the benign and 0.8485 for the malignant class. Both strategies based on transfer learning obtained higher recall when compared with the basic CNN approach. In the case of the F1-score, the proposed approach based on multi-level transfer learning has an F1-score of 0.9412 for the benign class and 0.9062 for the malignant class, while that of single-level transfer learning is 0.932 for the benign class and 0.8888 for the malignant class. Both approaches based on transfer learning obtained a higher F1-score when compared with the baseline approach, which uses regularization and data augmentation. The above results indicate that our method significantly outperforms the other two methods in all metrics. The performance of the pre-trained model for the thyroid nodule classification is also given in Table 7. The performance of the proposed model is considerably better than other methods.

### 5.4. Comparison of Different Pre-Trained CNN Architectures for the Classification of Thyroid Ultrasound Images

Table 8 provides the detailed experimental results for thyroid nodule characterization from thyroid ultrasound images. It indicates that, for the DDTI datasets, the Xception and Inception networks achieved the best accuracy, precision, and recall.

### 5.5. Receiver Operating Characteristics Curve

**Figure 16:**
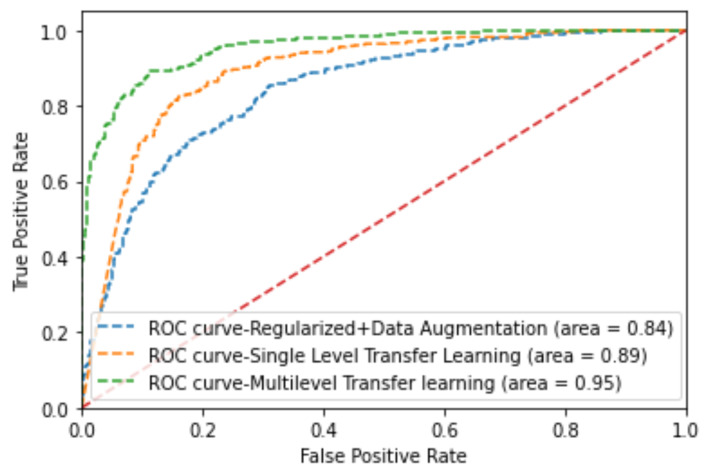
ROC analysis of the proposed models for DDTI dataset with 60–40% training and testing set splitting in 10-fold cross validation average).

In addition, we plotted ROC curves for each model in Figure 16, along with the AUC value for each model. It visualizes the trade-off between True Positive Rate (TPR) and False Positive Rate (FPR). This figure illustrates how much difference single-level and multi-level transfer learning can make. As shown in Figure 16, the ROC curve of the multi-level transfer learning method is close to the upper-left corner compared with the other two techniques. We can quantify the ROC curve to evaluate the performance of the models further with the AUC value as shown in Figure 16. The AUC value of the model, which follows the multi-level transfer learning technique, has a higher AUC value than the other two methods. Compared with the model without transfer learning, the models that use transfer learning exhibit improved performance, as indicated in Table 7 and Figure 16. Likewise, compared with single-level transfer learning, more improvement can be seen in multi-level transfer learning techniques, which use a medical dataset as a bridge dataset.

### 5.6. Comparison with the State of the Art Methods

Previous studies have used CNN models to diagnose thyroid cancer in the past, but the samples were small and their accuracy was not significant. As our results have demonstrated, incorporating squeeze and excitation module in the inception architecture along with the application of multi-level transfer learning improved the accuracy, sensitivity, specificity, and AUC of the proposed model.

In the experiment analysis, we used the public dataset, DDTI, which includes thyroid nodules with varying sizes, shapes, textures, and locations. A large number of published works rely on private datasets which cannot be used for experimentation. Therefore, comparing the performance of our approach with these existing approaches is difficult. We have tabulated the performance of various models taken from the literature, and it is shown in Table 9.

### 5.7. Advantages

The proposed deep-learning approach for classifying thyroid nodules could contribute to clinical practice in different ways. Predictions made by radiologists can differ depending on the individual level of experience and expertise [4]. This automated deep-learning solution can significantly reduce image interpretation time in clinical practice and can provide more accurate results. The readout time for the model was roughly 1.15 s per image. By contrast, the radiologists took approximately 30–40 s to classify one thyroid ultrasound image [4]. Finally, the changes adopted in the improved inception network structure are not only applicable to Inception networks but are also suitable for any convolutional neural network architecture such as residual network and densenet architectures. It is worth mentioning that the approach does not increase the depth of the neural network, and it is easy to deploy. The proposed network architecture is applicable to any image classification domain.

### 5.8. Limitations and Future Scope

It is important to emphasize that our study has several limitations. The lack of sufficient annotated thyroid ultrasound images has been a predicament in the computer-aided detection and characterization of thyroid nodules. A large dataset is required for the development of an efficient CAD system for thyroid nodule diagnosis. To implement and validate a new CAD system, it is necessary to use large datasets [57]. However, this poses a considerable barrier to utilizing the capability of deep learning concepts [57]. Even publicly available datasets of thyroid ultrasound images with manual annotations exist; the number of thyroid cases is limited to hundreds. Collecting a large, comprehensive dataset is required to develop effective CAD systems using deep learning techniques. As a pilot study, our analysis revolves around a public dataset with limited samples drawn from a retrospective and single-center study. Even though a different augmentation approach had to be used to enlarge the sample size, the issues related to the small sample size must be solved. Single-level and multi-level transfer learning have been utilized to address small sample size issues. In multi-level transfer learning techniques, a breast ultrasound image dataset was used as a bridge dataset, consisting of 1200 images. In the future, we will incorporate a dataset containing more samples as a bridge dataset. The proposed approach centered on the presumption that each image included one nodule. In the image where the sonographer delineated two nodules, we divided the image so that only one nodule could be seen. This research has focused only on developing a computer-aided characterization tool to classify benign and malignant thyroid nodules in thyroid ultrasound images. In the future, several aspects must be explored to improve accuracy, performance, and clinical applicability. We suggest a few directions and challenges for future research into thyroid image analysis. In future work, we intend to refine our detection and characterization framework. We can incorporate a detection network into the study that semantically segments the thyroid nodules from thyroid ultrasound images for better thyroid nodule diagnosis. It will provide physicians with a more comprehensive diagnostic model that aids them in risk evaluation and characterization. Furthermore, inception blocks can be replaced with dense or residual blocks. This approach is helpful for clinicians dealing with low-contrast images or images with uneven contrast ratios.

## 6. Conclusions

This paper mainly explores the effectiveness of squeeze and excitation networks and parallel convolutions in the inception architecture to characterize thyroid nodules. We also utilized a multi-level transfer learning technique that uses a bridge dataset from the same domain(ultrasound imaging) as the target domain to address limited sample size issues. The domain difference between the source and target domain is a major concern in single-level transfer learning. These models exhibited better diagnostic performance than state-of-the-art models. Based on the performance of different convolutional neural network models, the proposed approach can significantly improve the diagnosing capability of CAD systems for thyroid nodules. Furthermore, the model represents a generalized platform that can assist clinicians working across multiple domains.

## Figures and Tables

**Table 1:** Details of the breast and thyroid ultrasound image datasets.

Phase	Phase 2	Phase 3
# of Images	1312	980
Class distribution	(891 B, 421 M)	(658 B, 322 M)
Training	1004	750
Testing	111	83
Validation	197	147

**Table 2:** Various hyperparameters and their value for the training.

Parameter	Value
Image size	256×256
convolution layer filters	[16, 32, 64, 128, 256, 512]
convolution-layer,kernel size, stride,padding	3 × 3, 1 × 1, same
Activation function	Relu
Maxpooling: pool size, stride, padding	2 × 2, 2 × 2, same
Depth	5
Dropout	0.3
Batch size	32
Activation Function	Sigmoid
Number of epochs	100
Cross Validation	Stratified 5-Fold

**Table 3:** Parameter Settings for Data Augmentation.

Parameters of Image Augmentation	Values
Zoom range	0.2
Rotation range	20
width shift range	0.2
height shift range	0.2
feature-wise center	True
feature-wise standard normalization	True
horizontal flip	True
validation split	0.2
fill-mode	reflect

**Table 4:** Performance of the Network during Phase 1 and Phase 2.

Phase	Accuracy	Precision	Recall	F1-Score
Phase 1	0.9857	0.9790	0.9286	0.8975
Phase 2	0.8967	0.8567	0.9286	0.8340

**Table 5:** Detailed Evaluation of the performance of different Thyroid Nodule Ultrasound Classification models.

Method	Category	Precision	Recall	F1-Score
Hybrid Inception-SE (without TL)	Benign	0.8868	0.9592	0.9216
	Malignant	0.9333	0.8235	0.8750
Hybrid Inception-SE (with sL-TL)	Benign	0.9000	0.9600	0.932
	Malignant	0.9333	0.8485	0.8888
Hybrid Inception-SE (with mL-TL)	Benign	0.9057	0.9796	0.9412
	Malignant	0.9667	0.8529	0.9062
Inception-V3	Benign	0.8868	0.9216	0.9038
	Malignant	0.8667	0.8125	0.8387
Inception-ResNet v2	Benign	0.8868	0.9400	0.9126
	Malignant	0.9000	0.8182	0.8571

**Table 6:** Detailed Evaluation of the performance of different Thyroid Nodule Ultrasound Classification models.

Method	Category	G-Mean	Specificity
Hybrid Inceptionv3 (without TL)	Benign	0.8888	0.8235
	Malignant	0.8888	0.9592
Hybrid Inceptionv3 (with sL-TL)	Benign	0.9025	0.8485
	Malignant	0.9025	0.9600
Hybrid Inceptionv3 (with mL-TL)	Benign	0.9141	0.8529
	Malignant	0.9141	0.9796
Inception-v3	Benign	0.8653	0.8125
	Malignant	0.8653	0.9216
Inception-ResNet v2	Benign	0.8770	0.8182
	Malignant	0.8770	0.9400

**Table 7:** Detailed Evaluation of the performance of different Thyroid Nodule Ultrasound Classification models.

Method	Specificity	F1-Score	G-Mean	Sensitivityy	Accuracy
Hybrid Inception-v3 (without TL)	0.8913	0.8982	0.8888	0.8913	0.9036
Hybrid Inception-v3 (with sL-TL)	0.9042	0.9104	0.9025	0.9042	0.9156
Hybrid Inception-v3 (with mL-TL)	0.9162	0.9237	0.9140	0.9162	0.9277
Inception-V3	0.8670	0.8713	0.8653	0.8670	0.8795
Inception-ResNet v2	0.8791	0.8849	0.8770	0.8791	0.8916

**Table 8:** Comparison of various CNN models in the Binary Classification task.

Model	Specificity	F1-Score	G-Mean	Sensitivity	Accuracy
VGG8	0.7650	0.7651	0.7623	0.7651	0.7831
VGG11	0.7780	0.7798	0.7749	0.7779	0.7952
VGG16	0.7912	0.7912	0.7890	0.7912	0.8072
VGG19	0.8037	0.8056	0.8013	0.8037	0.8193
ResNet10	0.8172	0.8173	0.8157	0.8173	0.8313
ResNet18	0.8037	0.8056	0.8013	0.8037	0.8193
ResNet50	0.8162	0.8198	0.8135	0.8162	0.8313
DenseNet121	0.8416	0.8455	0.8394	0.8416	0.8554
Xception	0.8551	0.8575	0.8538	0.8551	0.8675
Inception-v3	0.8670	0.8713	0.8653	0.8670	0.8795
InceptionResNet-v2	0.8791	0.8849	0.8770	0.8791	0.8916

**Table 9:** Performance analysis of state-of-the-art thyroid nodule characterization methods for 2D ultrasound images.

Methods	Benign	Malignant	Accuracy	Sensitivity	Specificity	AUC
Chang et al. [54]	403	207	0.9200	100	0.8788	NG
Acharya et al. [15]	651	386	0.9310	0.9080	0.9450	0.9770
Nam et al. [55]	2450	2557	0.9050	0.9376	0.9139	0.9091
Sun et al. [56]	400	400	0.8825	0.9000	0.8650	0.9286
Wang et al. [22]	69	86	0.9680	0.9821	0.9565	NG
Liu et al. [16]	2551	5139	0.971	0.982	0.951	NG
Liu et al. [16]	128	322	0.928	0.965	0.780	NG
Chi et al. [18]	71	357	0.9829	0.9910	0.9390	NG
Y.Chen et al. [24]	552	602	92.11	NG	NG	NG

## Data Availability

Not applicable.

## References

[1] Mourad M., Moubayed S., Dezube A., Mourad Y., Park K., Torreblanca-Zanca A., Torrecilla J., Cancilla J., Wang J. (2020). Machine Learning and Feature Selection Applied to SEER Data to Reliably Assess Thyroid Cancer Prognosis. Sci. Rep..

[2] Park Y., Lee B.J. (2021). Machine learning-based prediction model using clinico-pathologic factors for papillary thyroid carcinoma recurrence. Sci. Rep..

[3] Li L., Xu M., Liu H., Li Y., Wang X., Jiang L., Wang Z., Fan X., Wang N. (2020). A Large-Scale Database and a CNN Model for Attention-Based Glaucoma Detection. IEEE Trans. Med. Imaging.

[4] Zhu Y.C., AlZoubi A., Jassim S., Jiang Q., Zhang Y., Wang Y.B., Ye X.D., Du H. (2021). A generic deep learning framework to classify thyroid and breast lesions in ultrasound images. Ultrasonics.

[5] Lal S., Das D., Alabhya K., Kanfade A., Kumar A., Kini J. (2021). NucleiSegNet: Robust deep learning architecture for the nuclei segmentation of liver cancer histopathology images. Comput. Biol. Med..

[6] Wang Y., Guan Q., Lao I., Wang L., Wu Y., Li D., Ji Q., Wang Y., Zhu Y., Lu H. (2019). Using deep convolutional neural networks for multi-classification of thyroid tumor by histopathology: A large-scale pilot study. Ann. Transl. Med..

[7] Szegedy C., Liu W., Jia Y., Sermanet P., Reed S., Anguelov D., Erhan D., Vanhoucke V., Rabinovich A. Going deeper with convolutions. Proceedings of the 2015 IEEE Conference on Computer Vision and Pattern Recognition (CVPR).

[8] Fernandez J. Attention in Computer Vision-Published in towards Data Science. https://towardsdatascience.com/attention-in-computer-vision-fd289a5bd7ad.

[9] Pröve P.L. Squeeze-and-Excitation Networks. https://towardsdatascience.com/squeeze-and-excitation-networks-9ef5e71eacd7.

[10] Olatunji S.O., Alotaibi S., Almutairi E., Alrabae Z., Almajid Y., Altabee R., Altassan M., Basheer Ahmed M.I., Farooqui M., Alhiyafi J. (2021). Early diagnosis of thyroid cancer diseases using computational intelligence techniques: A case study of a Saudi Arabian dataset. Comput. Biol. Med..

[11] Sharifi Y., Sargolzaei M., Bakhshali M.A., Dehghani T., Danaiashgzari M., Eslami S. (2020). Deep Learning on Ultrasound Images of Thyroid Nodules. Biocybern. Biomed. Eng..

[12] Keramidas E.G., Iakovidis D.K., Maroulis D., Dimitropoulos N. Thyroid texture representation via noise resistant image features. Proceedings of the 2008 21st IEEE International Symposium on Computer-Based Medical Systems.

[13] Tsantis S., Dimitropoulos N., Cavouras D., Nikiforidis G. (2009). Morphological and wavelet features towards sonographic thyroid nodules evaluation. Comput. Med. Imaging Graph..

[14] Singh N., Jindal A. (2012). Ultra sonogram images for thyroid segmentation and texture classification in diagnosis of malignant (cancerous) or benign (non-cancerous) nodules. Int. J. Eng. Innov. Technol..

[15] Acharya U.R., Sree S.V., Krishnan M.M.R., Molinari F., ZieleŸnik W., Bardales R.H., Witkowska A., Suri J.S. (2014). Computer-aided diagnostic system for detection of Hashimoto thyroiditis on ultrasound images from a Polish population. J. Ultrasound Med..

[16] Liu T., Guo Q., Lian C., Ren X., Liang S., Yu J., Niu L., Sun W., Shen D. (2019). Automated detection and classification of thyroid nodules in ultrasound images using clinical-knowledge-guided convolutional neural networks. Med. Image Anal..

[17] Ma J., Wu F., Zhu J., Xu D., Kong D. (2017). A pre-trained convolutional neural network based method for thyroid nodule diagnosis. Ultrasonics.

[18] Chi J., Walia E., Babyn P., Wang J., Groot G., Eramian M. (2017). Thyroid nodule classification in ultrasound images by fine-tuning deep convolutional neural network. J. Digit. Imaging.

[19] Gao L., Liu R., Jiang Y., Song W., Wang Y., Liu J., Wang J., Wu D., Li S., Hao A. (2018). Computer-aided system for diagnosing thyroid nodules on ultrasound: A comparison with radiologist-based clinical assessments. Head Neck.

[20] Song W., Li S., Liu J., Qin H., Zhang B., Zhang S., Hao A. (2018). Multitask cascade convolution neural networks for automatic thyroid nodule detection and recognition. IEEE J. Biomed. Health Inform..

[21] Li X., Zhang S., Zhang Q., Wei X., Pan Y., Zhao J., Xin X., Qin C., Wang X., Li J. (2019). Diagnosis of thyroid cancer using deep convolutional neural network models applied to sonographic images: A retrospective, multicohort, diagnostic study. Lancet Oncol..

[22] Wang L., Yang S., Yang S., Zhao C., Tian G., Gao Y., Chen Y., Lu Y. (2019). Automatic thyroid nodule recognition and diagnosis in ultrasound imaging with the YOLOv2 neural network. World J. Surg. Oncol..

[23] Wang J., Jiang J., Zhang D., Zhang Y.Z., Guo L.H., Jiang Y., Du S., Zhou Q. (2021). An integrated AI model to improve diagnostic accuracy of ultrasound and output known risk features in suspicious thyroid nodules. Eur. Radiol..

[24] Chen Y., Li D., Zhang X., Jin J., Shen Y. (2021). Computer aided diagnosis of thyroid nodules based on the devised small-datasets multi-view ensemble learning. Med. Image Anal..

[25] Sun J., Wu B., Zhao T., Gao L., Xie K., Lin T., Sui J., Li X., Wu X., Ni X. (2023). Classification for thyroid nodule using ViT with contrastive learning in ultrasound images. Comput. Biol. Med..

[26] (2023). Fusing enhanced Transformer and large kernel CNN for malignant thyroid nodule segmentation. Biomed. Signal Process. Control..

[27] Liang X., Yu J., Liao J., Chen Z. (2020). Convolutional Neural Network for Breast and Thyroid Nodules Diagnosis in Ultrasound Imaging. BioMed Res. Int..

[28] Raj B. A Simple Guide to the Versions of the Inception Network-Published in towards Data Science. https://towardsdatascience.com/a-simple-guide-to-the-versions-of-the-inception-network-7fc52b863202.

[29] Liu Z., Yang C., Huang J., Liu S., Zhuo Y., Lu X. (2021). Deep learning framework based on integration of S-Mask R-CNN and Inception-v3 for ultrasound image-aided diagnosis of prostate cancer. Future Gener. Comput. Syst..

[30] Szegedy C., Vanhoucke V., Ioffe S., Shlens J., Wojna Z. Rethinking the Inception Architecture for Computer Vision. Proceedings of the 2016 IEEE Conference on Computer Vision and Pattern Recognition (CVPR).

[31] Pereyra G., Tucker G., Chorowski J., Kaiser L., Hinton G.E. (2017). Regularizing Neural Networks by Penalizing Confident Output Distributions. arXiv.

[32] Szegedy C., Ioffe S., Vanhoucke V. (2016). Inception-v4, Inception-ResNet and the Impact of Residual Connections on Learning. arXiv.

[33] Szegedy C., Ioffe S., Vanhoucke V., Alemi A.A. Inception-v4, Inception-ResNet and the Impact of Residual Connections on Learning. Proceedings of the Thirty-First AAAI Conference on Artificial Intelligence, AAAI’17.

[34] Schlemper J., Oktay O., Chen L., Matthew J., Knight C.L., Kainz B., Glocker B., Rueckert D. (2018). Attention-Gated Networks for Improving Ultrasound Scan Plane Detection. arXiv.

[35] Hu J., Shen L., Sun G. (2017). Squeeze-and-Excitation Networks. arXiv.

[36] Cui Z., Gao Z., Leng J., Zhang T., Quan P., Zhao W. Alzheimer’s Disease Diagnosis Using Enhanced Inception Network Based on Brain Magnetic Resonance Image. Proceedings of the 2019 IEEE International Conference on Bioinformatics and Biomedicine (BIBM).

[37] Niu S., Liu M., Liu Y., Wang J., Song H. (2021). Distant Domain Transfer Learning for Medical Imaging. IEEE J. Biomed. Health Inform..

[38] Wen Y., Chen L., Deng Y., Zhou C. (2021). Rethinking pre-training on medical imaging. J. Vis. Commun. Image Represent..

[39] Kim H.G., Choi Y., Ro Y.M. Modality-bridge transfer learning for medical image classification. Proceedings of the 2017 10th International Congress on Image and Signal Processing, BioMedical Engineering and Informatics (CISP-BMEI).

[40] Alzubaidi L., Al-Amidie M., Al-Asadi A., Humaidi A.J., Al-Shamma O., Fadhel M.A., Zhang J., Santamaría J., Duan Y. (2021). Novel Transfer Learning Approach for Medical Imaging with Limited Labeled Data. Cancers.

[41] Mendes A., Togelius J., dos Santos Coelho L. (2020). Multi-Stage Transfer Learning with an Application to Selection Process. arXiv.

[42] Hung J.C., Chang J.W. (2021). Multi-level transfer learning for improving the performance of deep neural networks: Theory and practice from the tasks of facial emotion recognition and named entity recognition. Appl. Soft Comput..

[43] Lee J., Nishikawa R. (2020). Cross-Organ, Cross-Modality Transfer Learning: Feasibility Study for Segmentation and Classification. IEEE Access.

[44] Al-Dhabyani W., Gomaa M., Khaled H., Fahmy A. (2020). Dataset of breast ultrasound images. Data Brief.

[45] Krizhevsky A., Hinton G. (2009). Learning Multiple Layers of Features from Tiny Images. https://www.cs.toronto.edu/~kriz/learning-features-2009-TR.pdf.

[46] Le Y., Yang X. (2015). Tiny ImageNet Visual Recognition Challenge. https://github.com/seshuad/IMagenet.

[47] Milani P.M. (2017). The Power of Inception: Tackling the Tiny ImageNet Challenge. https://www.google.com/url?sa=t&rct=j&q=&esrc=s&source=web&cd=&ved=2ahUKEwir4tykm6aAAxWxt1YBHYsSAlYQFnoECCgQAQ&url=http%3A%2F%2Fcs231n.stanford.edu%2Freports%2F2017%2Fpdfs%2F928.pdf&usg=AOvVaw3NEeWleNYd7Fk74UoAia7W&opi=89978449.

[48] Agarwal D.P., Soni T.P., Sharma O.P., Sharma S. (2007). Synchronous malignancies of breast and thyroid gland: A case report and review of literature. J. Cancer Res. Ther..

[49] Turken O., NarIn Y., DemIrbas S., Onde M.E., Sayan O., KandemIr E.G., YaylacI M., Ozturk A. (2003). Breast cancer in association with thyroid disorders. Breast Cancer Res..

[50] Pedraza L., Vargas C., Narváez F., Durán O., Muñoz E., Romero E. An open access thyroid ultrasound image database. Proceedings of the 10th International Symposium on Medical Information Processing and Analysis.

[51] Ying X., Yu Z., Yu R., Li X., Yu M., Zhao M., Liu K. Thyroid Nodule Segmentation in Ultrasound Images Based on Cascaded Convolutional Neural Network. Proceedings of the 25th International Conference, ICONIP 2018.

[52] Nguyen D.T., Pham T.D., Batchuluun G., Yoon H.S., Park K.R. (2019). Artificial intelligence-based thyroid nodule classification using information from spatial and frequency domains. J. Clin. Med..

[53] Nguyen D., Kang J., Pham T., Batchuluun G., Park K. (2020). Ultrasound Image-Based Diagnosis of Malignant Thyroid Nodule Using Artificial Intelligence. Sensors.

[54] Chang C.Y., Lei Y.F., Tseng C.H., Shih S.R. (2010). Thyroid segmentation and volume estimation in ultrasound images. IEEE Trans. Biomed. Eng..

[55] Nam S.J., Yoo J., Lee H., Kim E.K., Moon H., Yoon J.H., Kwak J. (2016). Quantitative Evaluation for Differentiating Malignant and Benign Thyroid Nodules Using Histogram Analysis of Grayscale Sonograms. J. Ultrasound Med. Off. J. Am. Inst. Ultrasound Med..

[56] Liu T., Xie S., Yu J., Niu L., Sun W. Classification of thyroid nodules in ultrasound images using deep model based transfer learning and hybrid features. Proceedings of the 2017 IEEE International Conference on Acoustics, Speech and Signal Processing (ICASSP).

[57] Abdolali F., Shahroudnejad A., Hareendranathan A.R., Jaremko J.L., Noga M., Punithakumar K. (2020). A systematic review on the role of artificial intelligence in sonographic diagnosis of thyroid cancer: Past, present and future. arXiv.

